# Supplemental light application can improve the growth and development of strawberry plants under salinity and alkalinity stress conditions

**DOI:** 10.1038/s41598-022-12925-8

**Published:** 2022-06-03

**Authors:** Mohammad Reza Malekzadeh Shamsabad, Majid Esmaeilizadeh, Hamid Reza Roosta, Piotr Dąbrowski, Arkadiusz Telesiński, Hazem M. Kalaji

**Affiliations:** 1 Department of Horticultural Sciences, Faculty of Agriculture, Vali-e-Asr University of Rafsanjan, Kerman, 7718817111 Iran; 2 Department of Horticultural Sciences, Faculty of Agriculture and Natural Resources, Arak University, Arak, 38156-8-8349 Iran; 3 Department of Environmental Development, Institute of Environmental Engineering, Warsaw University of Life Sciences-SGGW, Nowoursynowska str. 159, 02-776 Warsaw, Poland; 4 Department of Bioengineering, West Pomeranian University of Technology in Szczecin, 17 Słowackiego Street, 71-434 Szczecin, Poland; 5 Department of Plant Physiology, Institute of Biology, Warsaw University of Life Science, 159 Nowoursynowska St., 02-776 Warsaw, Poland; 6 Institute of Technology and Life Sciences - National Research Institute, Falenty, Al. Hrabska 3, 05-090 Raszyn, Poland

**Keywords:** Abiotic, Light responses, Plant physiology

## Abstract

The use of complementary light spectra is a potential new approach to studying the increase in plant resilience under stress conditions. The purpose of this experiment was to investigate the effect of different spectra of complementary light on the growth and development of strawberry plants under salinity and alkalinity stress conditions. Plants were grown in the greenhouse under ambient light and irradiated with blue (460 nm), red (660 nm), blue/red (1:3), and white/yellow (400–700 nm) light during the developmental stages. The stress treatments were as follows: control (non-stress), alkalinity (40 mM NaHCO_3_), and salinity (80 mM NaCl). Our results showed that salinity and alkalinity stress decreased fresh and dry weights and the number of green leaves, and increased chlorotic, tip burn, and dry leaves. The blue and red spectra had a greater effect on reducing the effects of stress compared to other spectra. Stress conditions decreased SPAD and RWC, although blue light increased SPAD, and blue/red light increased RWC under stress conditions. Blue/red and white/yellow light had the greatest effect on reproductive traits. Stress conditions affected fruit color indicators, and red and blue light had the most significant effect on these traits. Under stress conditions, sodium uptake increased, while K, Ca, Mg, and Fe uptake decreased, markedly. Blue and red light and their combination alleviated this reducing effect of stress. It can be concluded that the effects of salinity and alkalinity stresses can be reduced by manipulating the supplemental light spectrum. The use of artificial light can be extended to stresses.

## Introduction

Various adverse environmental factors such as; salinity, alkalinity, drought, extreme temperatures, the toxicity of heavy metals, flooding, ultraviolet radiation, and ozone effect plants. Abiotic stresses disrupt the growth, physiology, and function of plants and are a very important challenge in crop production and food security^1^. Various strategies have been proposed to improve crop yield under stress, but most efforts to increase crop production in the presence of stressors have failed. It has always been tried to reduce the effects of these stresses on plants, and the use of different spectrums of complementary light can be considered.

Plant growth is controlled by the interaction of environmental and internal factors. Various environmental factors such as light, temperature, nutrients, and water play a significant role in strawberry plant growth. Plant response to light depends on factors such as light quality, environment, season, genotype, cultivation methods, etc.^2^. The quality of light depends on the composition of light and the effective wavelengths in photosynthesis, which has an important effect on the plant^3^. Therefore, light is an important energy source and an effective factor for growth, flowering, fruiting, and photosynthesis in plants^4^.

Initially, red LEDs (660 nm) were used with blue fluorescent lamps for lettuce, potato, spinach, and wheat in response to the need to develop better light sources for vertical and stratified crop growth systems^5^. Today, LED-based systems have been developed for plant physiology experiments^6^, suitable for research applications (in growth chamber)^7^, supplementation of lighting^8^, and adjustment of Photoperiod^9^. Artificial light and special light spectra in the greenhouse under stress conditions can be used to investigate the effects of specific spectra on plant tolerance under stress conditions.

In the past, greenhouse plants such as strawberries grew under natural light or artificial light such as fluorescent light. Recently, public interest in light-emitting diodes (LEDs) has increased^10^. Recently, The LED technology with specific wavelengths has been introduced and used as a complementary light source for crop cultivation. LED light technology has specific wavelengths and narrow^11^ bandwidth for plants. Reducing environmental impact is one of the most important strengths of selling and using artificial light sources^12^. A study showed that the environmental effects of using LED lighting technology are significantly less than fluorescent lamps. Therefore, the use of LED lighting in horticulture has many benefits, not only to improve product quality and save energy, but also to protect the water, soil, natural resources, and air/climate^13^. The effect of different wavelengths of LED light on different plants has been studied. It has been shown that LED radiation affects the nutrients and quality of strawberries^14^. Plant growth and physiology are strongly influenced by the light spectrum, which affects plant morphology, growth, and development^14^. The use of LED light in horticulture with specific wavelengths makes it possible to study the responses of plants to these wavelengths. Chl *a* and b are the main photosynthetic pigments in plants that absorb and use mainly blue and red wavelengths. The blue and red wavelengths absorbed by photosynthetic pigments in plants affect the efficiency of photosynthesis in plants^15^ and the biosynthesis of carotenoids^16^.

The effect of different light spectra on the biosynthesis of carotenoids is very significant due to their antioxidant activity in plants^16^. Red light increases transcription of the phytoene synthase (PSY) gene, which catalyzes the enzyme pathway for carotenoid biosynthesis^17^, and increases zeaxanthin concentration, facilitates stomata opening induced by blue light^18^. Manipulating the light spectrum using LED can increase the stress tolerance of plants by enhancing antioxidant compounds. So if the plant irradiates with blue and red lights, it will have optimal growth^19^ and higher stress tolerance. There are complex mechanisms by which plants respond to or tolerate stress^20^. Optimal plant growth is promoted by blue and red light and especially by their combination^19,21^. It has also been shown that the combination of blue and red lights under drought stress improves the physiological and morphological characteristics of lettuce^6^. Mirzahosseini et al.^22^ showed different defense strategies against wound stress in plants depending on the quality of light. They showed that Arabidopsis plants can better tolerate wound stress when exposed to a blue/red LED light source, mainly through a GA-independent signaling pathway, while white LED light triggers a GA-dependent stress response pathway. These studies should be expanded to provide a comprehensive understanding of the effects of light quality on plant responses to abiotic stress in other plants.

Environmental stresses significantly inhibit plant growth. Soil salinity and alkalinity are environmental stressors that have become a limiting factor for plant growth in agricultural production worldwide^23^. Previous experiments with LED light sources have attempted to improve plant growth and quality in a growth chamber or greenhouse^24,25^. Mickens et al.^26^ used different light spectra and simulated sunlight on pakchoi (*Brassica rapa* var. Chinensis). Another experiment compared the effects of different spectra of white, varied blue: red, and sunlight on lettuce^27^. However, using different spectrums of light as complementary light in the greenhouse may have positive effects on plants under salinity and alkalinity conditions. We can extend these experiments to stress conditions and study the response of plants to stress under different spectra of the LED light. Plants living under natural conditions are exposed to unfavorable factors that disrupt their growth and development, leading to reduced growth, development, and yield. The application of complementary light spectra using light-emitting diodes (LEDs) represents a potential new approach for studying the increasing resilience of plants under stress conditions.

We hypothesize that different wavelengths of complementary light in greenhouse conditions reduce the negative effects of salinity and alkalinity stresses by affecting the photosynthetic apparatus, vegetative and reproductive growth, and ions uptake. Since the cultivar Camarosa is one of the most stress-sensitive cultivars among strawberry cultivars, this study aimed to investigate the effects of different light spectra on the growth and development of the strawberry cv. Camarosa under salinity and alkalinity stress conditions. We investigated the vegetative and reproductive characteristics, leaf characteristics, and fruit color of the strawberry plants. We expect that the results of this experiment will improve the functional properties of complementary light and optimize the lighting strategies for strawberry plants. The application of LED technology in horticulture can be expanded in various fields. Most experiments are limited to the effect of some light spectra on plant growth and development. These studies can be extended to the effect of different light spectra on plants under stress conditions. Abiotic stresses such as salinity and alkalinity are widespread environmental problems and are the most severe hazards to agriculture^28^. The use of complementary light in greenhouses may also reduce the adverse effects of these stresses on greenhouse plants.

## Materials and methods

### Plant material and growth conditions

This experiment was conducted in the Vali-e-Asr University experimental greenhouse in 2020. We prepared rooted strawberry plants (*Fragaria* × *ananassa* Duch, cv. Camarosa) from a nursery in Karaj, Iran. Plants were planted in a hydroponic system in a 4-l pot containing cocopeat and perlite (ratio 70:30 V:V). Each treatment included three pots, and three plants were planted. The plants were irrigated with a pump and a drip. Irrigation of plants was done twice a day at 10 am and 3 pm. Each time irrigation, 150 ml of the nutrient solution was given to the plants. The plants were growing in a greenhouse with a temperature of 25/15 ± 2 °C (day/night), 13/11 h (light/dark) photoperiod, relative humidity of 50 ± 10%, and maximum light intensity above the canopy per day 1085 μmol m^−2^ s^−1^ (LED + ambient light). The plants were irrigated with Morgan nutrient solution^29^ (EC: 1.4 dS m^−1^, pH: 6.5) (Table 1). Plants were treated by five different light conditions (light spectrum) and three stress levels, including control (non-stress), alkalinity (40 mM NaHCO_3_), and salinity (80 mM NaCl). The alkalinity and salinity treatments were applied twenty days after planting. Saline and alkaline solutions were given 100 ml per pot every three days.

**Table 1:** Concentration of nutrients used in the nutrient solution of this experiment.

Macronutrients	Concentration (mg l^−1^)	Micronutrients	Concentration (mg l^−1^)
N	128	Fe	5
P	58	Mn	2
K	211	Zn	0.25
Ca	104	B	0.7
Mg	40	Cu	0.07
S	54	Mo	0.05

### LED tubes and the light treatments

**Figure 1:**
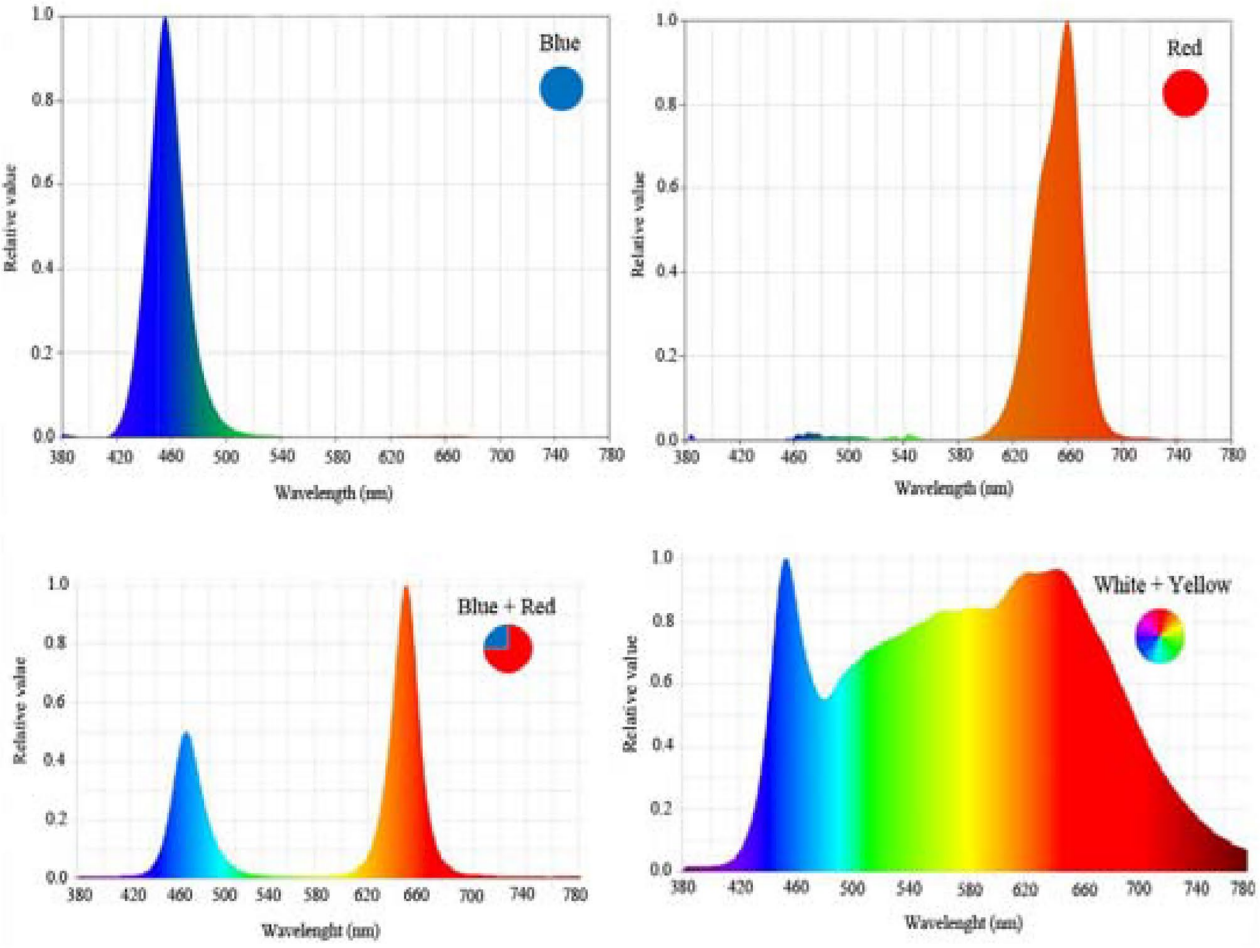
Relative distribution of different spectral LEDs (monochromatic blue, monochromatic red, blue/red (1:3), and white/yellow (1:1) used during plant growth.

Plants cultivated under metal structures (length: 100 cm, width: 5 cm, and height: 5 cm) With LED tubes with 24 W of power and photon flux density (PPFD) of 200 μmol m^−2^ s^−1^ (Parto Roshd Novin Company, Iran grow light, Iran) of different spectral ranges: monochromatic blue (B) (with peak 460 nm), monochromatic red (R) (with peak 660 nm), dichromatic blue/red (1:3), white/yellow (1:1) (400–700 nm) (Fig. 1) and only ambient light (Table 2). The photoperiod was 11 h of light and 13 h of darkness. Directly above each of the plants, LED lighting systems were mounted 30 cm apart, and the illumination intensities of the LEDs were maintained at 200 μmol m^−2^ s^−1^ at the leaf surface. LED + ambient light received by plants had a photon flux density of 1085 μmol m^−2^ s^−1^.

**Table 2:** Characteristics of LEDs used in this experiment.

Manufacture company	CRI	Number of LEDs	Light coverage area	Power consumption	Lens type	Certificate
Iran grow light	95%	24	40 cm × 100 cm	24 watts	90°	CCC, CE, FEC, Ip45, RoHS

### Vegetative growth and leaf characteristics

At the end of the experiment, plants were harvested for measurement. Their branches, roots, and crowns were separated. Samples were weighed, and fresh weight was recorded. To measure dry weight, the samples were placed in an oven at 70 °C for 72 h, and then the dry weight of the samples was recorded. The number of green, chlorotic, tip burned, and dry leaves for each treatment was recorded at the end of the experiment and before harvest. Petiole length was measured with a ruler. To measure leaf area, three-leaf samples were randomly collected from each treatment, and leaf area was measured with 202 m-CI leaf area.

### Leaf relative water content (LRWC) and SPAD values

The chlorophyll index in young leaves was recorded with a SPAD-502 Chlorophyll Meter (Minolta Camera Co. Ltd., Osaka, Japan). Three leaves were selected from each pot and measurements were made. Fresh leaves were used to determine the relative LRWC. One leaf from the fully expanded leaf was cut from each plant. Leaf disks (5 mm diameter) were obtained from the leaves. To determine the fresh weight (FW), the prepared leaf discs were weighed. Then, it floated on distilled water in a petri dish and incubated under normal room temperature. After four hours, the adhering water of the discs was blotted and then weighed to determine turgor weight (TW). The samples were dried at 70 °C for 24 h, and the dry weight (DW) was obtained. Relative water content was calculated using the following equation:

$$ \left( {{\text{LRWC in }}\% } \right) \, = \, \left[ {\left( {{\text{FW}} - {\text{DW}}} \right)/\left( {{\text{TW}} - {\text{DW}}} \right)} \right] \, \times { 1}00. $$

where FW: Fresh weight, DW: dry weight, TW: turgor weight.

### Reproductive characteristics and total soluble solids of fruit

Early yield, fruit number, fruit length, fruit diameter, and the number of inflorescences were measured during the growth period of plants. Total soluble solids (°Brix) of fruits were measured with a refractometer (PAL-1, Atago Co., Ltd; Japan).

### Fruit color

Fruit surface color was measured using a colorimeter (Chroma Meter CR-400C, Konica Minolta, Osaka, Japan). CIE L*a*b* coordinates were recorded: L* was lightness, a* (-greenness to‏ + redness), and b* (-blueness to + ‏yellowness) were the chromaticity coordinates^30^. Numerical values of a* and b* were converted into hue angle (H° = tan − 1 b*/a*) and chroma [Chroma = (a*2 + b*2)1/2]. Measurements were taken by reading three opposite points from the surface of the fruit.

### Elemental analysis

The dried leaf and root samples were exposed to 550 °C for 5 h, and the leaf samples were turned to ashes. The ashes were dissolved in 5 ml of 2 N HCl, and 50 ml of distilled water was added. Leaf and root Na and K concentrations were measured using flame photometry (Jenway, PFP7 model). Leaf and root iron were measured by atomic absorption spectrophotometer (model T80 UV/VIS made in China). Ca and Mg concentrations were measured by EDTA titration^31^.

### Experimental design and data analysis

This experiment was performed as a completely randomized design with two factors in three replications as factorial and three single plants in pots. SAS software version 9.4 was used for data analysis (SAS Institute, Cary, NC, USA. https://www.sas.com/en_us/home.html). All data were statistically analyzed using two-way ANOVA model. By observing significant treatment effects in the analysis of variance (ANOVA), significant mean differences (P < 0.05) were calculated using the multiple ranges Duncan test as a post hoc. Once the differences between the means are demonstrated, it is possible to determine which means are different using post hoc range tests and pairwise multiple comparisons. Range tests identify homogeneous subsets of means that do not different from each other. Principal component analysis and biplots were performed using XLSTAT software version 2015 (https://www.xlstat.com/en/news/version-2015-6). A correlation plot was drawn with Origin Pro software version 2021(https://www.originlab.com/2021). The graphs were drawn using Microsoft Excel (2016).

### Statement of compliance

The authors confirm that all the experimental research and field studies on strawberry plants, including the collection of plant material, complied with relevant institutional, national, and international guidelines and legislation.

## Results

### Vegetative characteristics

The results showed that light, stress and their interaction had significant effects on vegetative traits. Salinity and alkalinity stress caused significant reduction in fresh weight and dry weight of leaves, crowns and roots under all light conditions. Under salinity stress, blue light caused the least reduction in fresh leaves (− 27%), crowns (− 4%), and roots (− 15%) weight compared to the non-stress condition. Red light had the greatest effect on root fresh weight in salinity stress. Under alkaline stress conditions, blue light caused the least decrease in leaf fresh weight (− 52%) and crown (− 39%) compared to the control treatment. Red light had the greatest effect on root fresh weight and leaf, crown and root dry weight (Table 3) in alkalinity stress compared to control treatment.

**Table 3:** Interaction of light sources and stress (salinity and alkalinity) on vegetative characteristics of strawberry cv. Camarosa.

Light sources	Stress	Leaf fresh weight (g plant^−1^)	Crown fresh weight (g plant^−1^)	Root fresh weight (g plant^−1^)	Leaf dry weight (g plant^−1^)	Crown dry weight (g plant^−1^)	Root dry weight (g plant^−1^)
Blue (460 mn)	Control	23.1 ± 0.40^d^	6.22 ± 0.48^c^	33.2 ± 1.63^de^	6.44 ± 0.11^c^	1.15 ± 0.11^def^	4.33 ± 0.19^de^
	Salinity	17.4 ± 1.09^ef^	4.55 ± 0.29^cde^	26.7 ± 1.97^fg^	4.77 ± 0.11^de^	1.10 ± 0.11^def^	3.66 ± 0.19f.
	Alkalinity	11.0 ± 0.88^h^	3.77 ± 0.11^def^	17.5 ± 0.96^h^	2.77 ± 0.29^gh^	0.64 ± 0.10^i^	2.00 ± 0.19^g^
Red (660 nm)	Control	44.4 ± 2.42^a^	9.00 ± 0.96^b^	36.4 ± 0.55^cd^	8.22 ± 0.67^b^	1.52 ± 0.04^c^	4.66 ± 0.38^d^
	Salinity	17.1 ± 1.41^fg^	5.44 ± 0.11^cd^	31 ± 1.38^ef^	4.66 ± 0.19^de^	1.04 ± 0.07^efg^	3.88 ± 0.29^ef^
	Alkalinity	8.7 ± 0.48^hi^	5.22 ± 0.72^cd^	26.1 ± 1.74^g^	4.33 ± 0.50^def^	1.07 ± 0.05^d-g^	2.33 ± 0.02^g^
Blue/Red (1;3)	Control	37.3 ± 0.69^b^	9.66 ± 0.50^b^	50.0 ± 0.69^b^	10.30 ± 1.15^a^	1.82 ± 0.07^b^	6.44 ± 0.29^b^
	Salinity	17.8 ± 0.90^ef^	4.77 ± 0.67^cde^	38.3 ± 1.53^c^	4.33 ± 0.19^def^	0.94 ± 0.13^fgh^	4.77 ± 0.11^cd^
	Alkalinity	17.7 ± 0.29^ef^	3.00 ± 0.50^ef^	16.0 ± 1.83^h^	2.55 ± 0.29^h^	0.85 ± 0.06^ghi^	2.33 ± 0.19^g^
White/yellow (400–700 nm)	Control	44.0 ± 0.83^a^	11.50 ± 0.80^a^	68.4 ± 1.94^a^	10.20 ± 0.40^a^	2.28 ± 0.03^a^	8.77 ± 0.22^a^
	Salinity	20.1 ± 0.77^e^	5.66 ± 0.19^c^	37.5 ± 2.73^cd^	5.55 ± 0.40^cd^	1.21 ± 0.03^de^	5.33 ± 0.19^c^
	Alkalinity	14.2 ± 0.58^g^	4.66 ± 0.10^cde^	25.4 ± 0.80^g^	4.00 ± 0.50^efg^	1.15 ± 0.04^def^	3.44 ± 0.11^f^
Ambient light	Control	29.0 ± 1.20^c^	8.44 ± 1.44^b^	65.1 ± 2.29^a^	8.00 ± 0.69^b^	1.29 ± 0.07^d^	8.33 ± 0.19^a^
	Salinity	11.0 ± 0.66^h^	3.66 ± 0.38^def^	30.0 ± 0.96^efg^	3.22 ± 0.29^fgh^	0.75 ± 0.05^hi^	3.55 ± 0.29^f^
	Alkalinity	5.8 ± 0.58^i^	2.44 ± 0.29^f^	16.4 ± 1.05^h^	1.88 ± 0.40^h^	0.69 ± 0.05^i^	2.55 ± 0.22^g^
Significance	Light (L)	***	***	***	***	***	***
	Stress (S)	***	***	***	***	***	***
	L × S	***	*	***	**	***	***

Values are means ± SE of three replicates. Bars with different letters show significant differences at P ≤ 0.05 (Duncan).

Significance according to ANOVA, ns, *, **, ***, no significant and significant P ≤ 0.05, 0.01, 0.001, respectively.

Control (no stress), salinity (80 mM NaCl) and alkalinity (40 mM NaHCO_3_).

Photon flux density (PPFD) 200 ± 10 mmol_m^–2^ s^–1^.

SAS software version 9.4 was used for data analysis.

### Effect of stress and light spectrum on leaves

**Figure 2:**
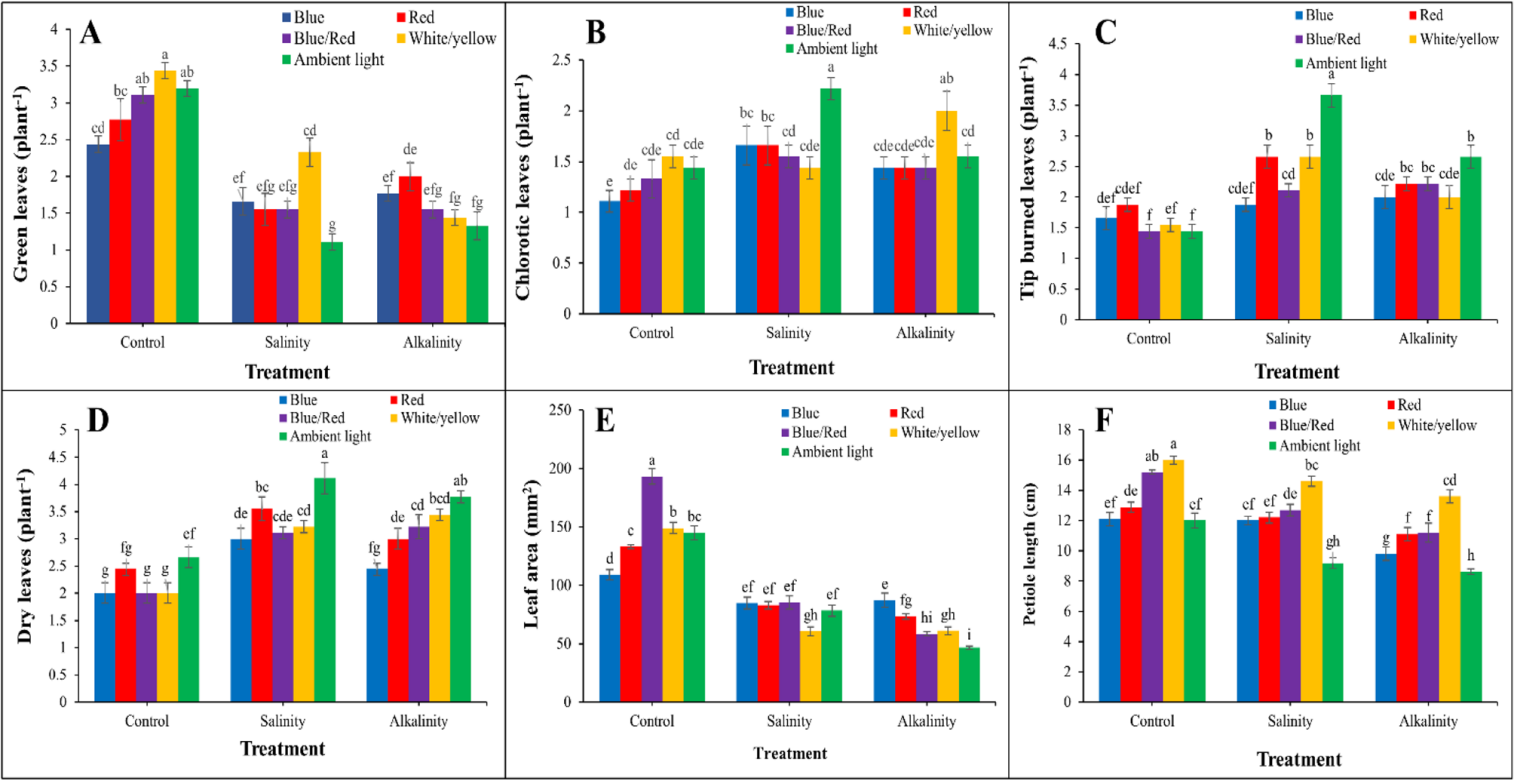
Changes in (**A**) green leaves; (**B**) chlorotic leaves; (**C**) tip burned leaves; (**D**) dry leaves; (**E**) leaf area; (**F**) petiole length under the effect of five light spectrum levels and three stress levels in three repetitions in strawberries cv. Camarosa. Means followed by the same letter for a parameter, are not significantly different according to Duncan (P ≤ 0.05). Vertical bars indicate the standard errors of three replicates. SAS software version 9.4 was used for data analysis. The graphs were drawn using Microsoft Excel (2016).

According to the results in Fig. 2, salt and alkalinity stress caused a significant decrease in the number of green leaves, leaf area, and petiole length, and an increase in chlorotic, tip burn, and dry leaves. The highest number of green leaves was observed in white/yellow light and without stress (control) treatment. Blue light under salt stress and red light under alkaline stress conditions had the lowest decrease in green leaves compared to the control treatment (Fig. 2A). The highest amount of chlorosis, burnt tips, and dry leaves were observed under stress and ambient light. Under salinity stress, the least amount of chloroses leaves was observed under white/yellow light (7%) and under alkaline stress conditions under blue/red light (8%) compared to the control treatment (Fig. 2B). Under salt stress, the least amount of tip burned and dry leaves were observed under blue light compared to other light spectrum treatments. Under alkaline stress conditions, blue light had the least amount of burned leaf tips. Red and blue light had the least amount of dry leaves compared to the control treatment (Fig. 2C,D). Under salinity and alkalinity stress, the highest leaf area was recorded under blue/red and blue light, respectively (Fig. 2E). Under salinity and alkaline stress, white/yellow light had the greatest effect on leaf petiole length (Fig. 2F).

### Leaf relative water content (LRWC) and SPAD values

**Figure 3:**
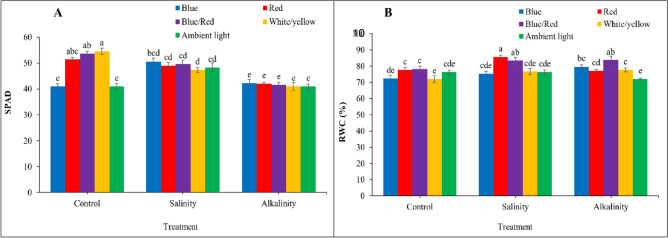
Changes in SPAD (**A**) and RWC (**B**) index under the effect of five light spectrum levels and three stress levels in three repetitions in strawberries cv. Camarosa. Means followed by the same letter for a parameter, are not significantly different according to the Duncan (P ≤ 0.05). Vertical bars indicate the standard errors of three replicates. SAS software version 9.4 was used for data analysis. The graphs were drawn using Microsoft Excel (2016).

According to the results (Fig. 3), light and stress had a significant effect on SPAD index and relative water content. The results showed that the SPAD index was highest under white/yellow, blue/red, and red light and decreased under salt and alkalinity stress conditions. Complementary blue light and ambient light under salt stress conditions increased the SPAD index compared with the control. For other light spectrums, the SPAD index decreased under stress conditions. The lowest SPAD index was observed under alkaline stress, and the light treatments did not differ significantly (Fig. 3A). Complementary light spectra were able to increase RWC under stress conditions compared to the control. Under salinity stress, the highest RWC was observed with red and blue/red light, while the other complementary light spectra were not significantly different. Under alkaline stress conditions, blue/red light had the greatest effect on RWC, and the lowest RWC was observed under ambient light (Fig. 3B).

### Reproductive characteristics and fruit soluble solids

The highest number of inflorescences and fruits was obtained in blue/red light treatment and control. Under salinity and alkali stress conditions, blue/red light caused a significant increase in inflorescences and fruits compared to other light spectra. Under salinity and alkalinity stress conditions, the highest early yield was observed in red and white/yellow light, respectively. Under salinity stress, blue/red light, and alkaline stress, white/yellow light caused the greatest increase in fruit length and diameter. The highest soluble solids content in fruit was observed under blue/red light and the lowest under non-supplemented light treatment (ambient light). According to our results, blue/red light had the greatest effect on inflorescence and fruit set, and white/yellow light had the greatest effect on fruit size and yield. Stress conditions reduced inflorescence, fruit number, and fruit size (Table 4).

**Table 4:** Interaction of five levels of light spectrum and three levels of stress treatment on reproductive characteristics of strawberry cv. Camarosa.

Light sources	Stress	Inflorescence number (Plant^−1^)	Early yield (g plant^−1^)	Fruit number (Plant^−1^)	Fruit length (mm)	Fruit diameter (mm)	Soluble solids (°Brix)
Blue (460 nm)	Control	2.11 ± 0.11^cd^	26.2 ± 0.9^fgh^	1.44 ± 0.11^fg^	36.0 ± 1.00^cd^	31.6 ± 1.66^bcd^	5.60 ± 0.66^f^
	Salinity	1.22 ± 0.11^g^	18.4 ± 1.09^i^	2.22 ± 0.11^efg^	31.6 ± 1.66^de^	25.0 ± 2.88^def^	7.83 ± 0.17^bcd^
	Alkalinity	1.22 ± 0.11^g^	25.6 ± 0.33^gh^	3.55 ± 0.29^cd^	30.0 ± 0.90^def^	30.0 ± 2.88^bcd^	8.76 ± 0.82^b^
Red (660 nm)	Control	2.66 ± 0.19^b^	48.0 ± 1.64^c^	5.77 ± 0.44^b^	40.0 ± 2.88^abc^	33.3 ± 3.33^bc^	7.13 ± 0.77^de^
	Salinity	1.66 ± 0.19^ef^	31.4 ± 2.37^e^	3.77 ± 0.61^cd^	32.0 ± 1.00^d^	26.6 ± 1.66^c-f^	7.30 ± 0.32^cde^
	Alkalinity	1.44 ± 0.11^fg^	25.2 ± 2.47^h^	2.00 ± 0.19^efg^	25.0 ± 2.88^ef^	25.0 ± 2.88^def^	7.30 ± 0.41^cde^
Blue/Red (1;3)	Control	3.77 ± 0.11^a^	68.2 ± 1.72^b^	9.22 ± 0.88^a^	43.3 ± 1.66^ab^	36.6 ± 1.66^b^	8.90 ± 0.26^b^
	Salinity	2.44 ± 0.11^bc^	29.6 ± 0.88^efg^	4.11 ± 0.22^c^	36.6 ± 3.30^bcd^	28.3 ± 1.66^cde^	12.20 ± 0.39^a^
	Alkalinity	1.55 ± 0.11^efg^	9.44 ± 0.601^j^	6.33 ± 0.57^b^	31.6 ± 1.66^de^	26.6 ± 1.66^c–f^	12.30 ± 0.16^a^
White/yellow (400–700)	Control	1.77 ± 0.11^def^	108.0 ± 1.07^a^	4.00 ± 0.33^c^	45.0 ± 2.88^a^	45.0 ± 2.88^a^	5.93 ± 0.84^ef^
	Salinity	1.88 ± 0.11^de^	30.3 ± 1.20^ef^	3.88 ± 0.11^cd^	30.0 ± 2.88^def^	28.3 ± 3.33^cde^	8.60 ± 0.11^bc^
	Alkalinity	1.44 ± 0.11^fg^	39.5 ± 2.37^d^	2.88 ± 0.22^de^	36.0 ± 1.00^cd^	30.0 ± 2.88^bcd^	7.80 ± 0.51^bcd^
Ambient light	Control	1.55 ± 0.11^fg^	27.1 ± 1.63^e–h^	2.44 ± 0.22^ef^	35.0 ± 2.88^cd^	26.6 ± 1.66^c–f^	5.43 ± 0.29^f^
	Salinity	1.44 ± 0.22^fg^	14.1 ± 0.86^i^	1.44 ± 0.11^fg^	23.3 ± 3.33^f^	21.6 ± 4.40^ef^	6.06 ± 0.29^ef^
	Alkalinity	1.44 ± 0.11^fg^	17.5 ± 1.44^i^	1.22 ± 0.11^g^	23.3 ± 3.33^f^	20.0 ± 0.90^f^	7.60 ± 0.20^bcd^
Significance	Light (L)	***	***	***	***	***	***
	Stress (S)	***	***	***	***	***	***
	L × S	***	***	***	ns	ns	**

Values are means ± SE of three replicates. Bars with different letters show significant differences at P ≤ 0.05 (Duncan).

Significance according to ANOVA, ns, *, **, ***, no significant and significant P ≤ 0.05, 0.01, 0.001, respectively.

Control (no stress), salinity (80 mM NaCl) and alkalinity (40 mM NaHCO_3_).

Photon flux density (PPFD) 200 ± 10 mmol_m^–2^ s^–1^.

SAS software version 9.4 was used for data analysis.

### Fruit color

The L* index indicates the luminosity of the fruit. The a index goes from + 60 (red) to − 60 (green) and decreases towards the center of the color diagram (gray, zero). The croma index determines the intensity or purity of the color. A change in the hue angle indicates a change in fruit color. An increase in the hue angle of strawberry fruit indicates that the color of the fruit changes from dark red to pinkish red^30^. Light and stress had a significant effect on fruit color parameters. Under stress conditions, the a-index increased, so that the highest value of a-index was recorded under salt and bicarbonate stresses and in the red-light spectrum. The stress conditions reduced the L-index, so that the highest L-index was measured in the control treatments. The highest L-index in salinity and alkalinity stress was measured in the blue/red and ambient light spectrum, respectively. The highest Croma index was measured in alkalinity stress and blue/red light spectrum. The lowest Croma index was measured in blue light and control treatment. The highest Hue index was observed in white/yellow light and control treatment, and the lowest in white/yellow light and salinity treatment. Under salinity and alkalinity stress, blue light had the highest hue index and white/yellow light had the lowest hue index compared to the other light spectra (Table 5).

**Table 5:** Interaction of five levels of light spectrum and three levels of stress treatment on fruit color parameters of strawberry cv. Camarosa.

Light sources	Stress	a	L*	Croma	Hue
Blue (460 nm)	Control	31.3 ± 0.14^fg^	46.2 ± 0.66^a^	38.5 ± 0.61^i^	39.5 ± 0.43^efg^
	Salinity	33.1 ± 0.60^c–f^	44.6 ± 0.89^abc^	44.3 ± 0.42^d–g^	46.1 ± 0.24^bc^
	Alkalinity	33.8 ± 0.40^b–e^	41.0 ± 1.21^fg^	49.3 ± 0.20^a^	51.4 ± 0.50^a^
Red (660 nm)	Control	32.5 ± 0.44^def^	46.3 ± 1.28^a^	45.1 ± 0.52^c–f^	48.6 ± 2.15^ab^
	Salinity	35.4 ± 0.49^abc^	40.0 ± 0.28^gh^	42.5 ± 0.81^gh^	36.9 ± 3.46^gh^
	Alkalinity	37.5 ± 0.34^a^	41.7 ± 1.00^efg^	47.8 ± 0.28^ab^	42.6 ± 0.61^c–f^
Blue/Red (1;3)	Control	33.7 ± 0.46^b–f^	45.6 ± 0.66^ab^	45.6 ± 0.45^cde^	47.0 ± 0.97^abc^
	Salinity	35.2 ± 0.50^a–d^	44.8 ± 1.00^abc^	45.9 ± 0.37^bcd^	44.5 ± 0.43^bcd^
	Alkalinity	36.3 ± 1.88^ab^	38.5 ± 0.25^h^	46.8 ± 0.60^bc^	43.8 ± 2.00^b–e^
White/yellow (400–700)	Control	29.4 ± 0.9^g^	44.2 ± 0.54^a–d^	43.0 ± 0.84^fgh^	51.5 ± 2.00^a^
	Salinity	34.2 ± 0.61^b–f^	42.0 ± 0.55^d–g^	43.6 ± 0.38^efg^	33.9 ± 2.86^h^
	Alkalinity	34.8 ± 1.17^a–e^	38.2 ± 1.24^h^	42.2 ± 1.02^gh^	38.3 ± 1.28^fgh^
Ambient light	Control	31.5 ± 1.77^fg^	46.6 ± 0.41^a^	41.4 ± 1.61^h^	44.8 ± 1.21^bcd^
	Salinity	33.7 ± 1.90^b–f^	42.4 ± 0.73^c–f^	42.3 ± 0.89^gh^	41.2 ± 0.83^d–g^
	Alkalinity	32.1 ± 0.71^efg^	43.5 ± 0.72^b–e^	41.4 ± 0.71^h^	43.2 ± 2.08^cde^
Significance	Light (L)	**	**	***	**
	Stress (S)	***	***	***	***
	L × S	ns	**	***	***

Values are means ± SE of three replicates. Bars with different letters show significant differences at P ≤ 0.05 (Duncan).

Significance according to ANOVA, ns, *, **, ***, no significant and significant P ≤ 0.05, 0.01, 0.001, respectively.

Control (no stress), salinity (80 mM NaCl) and alkalinity (40 mM NaHCO_3_).

Photon flux density (PPFD) 200 ± 10 mmol_m^–2^ s^–1^.

SAS software version 9.4 was used for data analysis.

### Elemental analysis

Table 6 shows that light and stress have a significant effect on root and shoot elements concentrations. Salinity and alkalinity stress increased Na content in shoot and root. The highest Na content was observed in the salinity and ambient light treatments. The lowest Na contents in shoot and roots were observed under blue/red and white/yellow light, respectively. Under salinity stress, the lowest Na content in shoot and root was observed under blue light. Under alkaline conditions, the lowest Na content in shoot and root was observed under blue and red light, respectively. Plants grown under red light and without stress had the highest amount of shoot and root K. Under salt stress, plants treated with red light had the highest amount of shoot and root K. Under alkaline stress conditions, the highest amount of shoot and root K was obtained under blue/red and red light, respectively. Under alkaline stress conditions, blue/red light increased shoot K content, and under salt stress conditions, blue and blue/red light increased root K content compared with the control treatment. Under other light conditions, K content decreased compared with the control. Salt and alkalinity stress decreased Ca, Mg, and Fe content in shoot and root. The highest Ca and Fe content in shoot and roots and the highest Mg content in the shoot were observed under blue/red light, and the highest Mg content in roots was observed under blue light. Under stress conditions, plants grown under blue/red and red light had the highest Ca and Mg contents in the shoot. The blue light had the greatest effect on Ca and Mg in roots. The highest Fe content in shoot and root was found in plants grown under blue/red light (Table 6).

**Table 6:** Interaction of five levels of light spectrum and three levels of stress treatment on nutrient element concentration of strawberry cv. Camarosa.

Light sources	Stress	Na (%DW)		K (%DW)		Ca (%DW)		Mg (%DW)		Fe (mg kg^−1^DW)	
		Shoot	Root	Shoot	Root	Shoot	Root	Shoot	Root	Shoot	Root
Blue (460 nm)	Control	2.96^de^	3.05^de^	2.81^a–e^	1.77^abc^	0.130^ab^	0.150^b^	0.117^b^	0.170^a^	81.3^b^	84.0^dc^
	Salinity	3.15^cde^	3.77^bcd^	2.77^a–e^	1.80^abc^	0.096^e^	0.116^cd^	0.111^b–e^	0.153^bc^	57.6^ef^	71.0^fg^
	Alkalinity	2.99^de^	4.37^ab^	2.54^b–f^	1.60^bcd^	0.123^abc^	0.133^bc^	0.107^c–f^	0.138^de^	52.3^fgh^	77.0^ef^
Red (660 nm)	Control	3.12^cde^	3.00^e^	3.14^a^	2.00^a^	0.126^abc^	0.183^a^	0.144^a^	0.154^b^	65.6^d^	95.0^ab^
	Salinity	3.27^cde^	3.96^bc^	3.04^ab^	1.84^abc^	0.123^abc^	0.150^b^	0.095^gh^	0.132^def^	62.0^de^	81.0^de^
	Alkalinity	3.21^cde^	3.87^bc^	2.34^def^	1.9^ab^	0.116^bcd^	0.120^cd^	0.135^a^	0.125^fd^	54.0^fg^	67.3^gh^
Blue/Red (1;3)	Control	2.62^e^	3.09^de^	2.40^c-f^	1.46^cde^	0.136^a^	0.180^a^	0.140^a^	0.142^cd^	88.6^a^	98.0^a^
	Salinity	3.40^bcd^	4.21^ab^	2.37^c-f^	1.73^abc^	0.123^abc^	0.120^cd^	0.116^bc^	0.124^fg^	73.6^c^	83.0^cde^
	Alkalinity	3.15^cde^	3.99^bc^	2.94^abc^	1.16^e^	0.123^abc^	0.100^de^	0.114^bcd^	0.114^gh^	75.0^bc^	84.6^cd^
White/yellow (400–700)	Control	2.71^de^	2.80^e^	2.84^a–d^	1.93^ab^	0.130^ab^	0.146^b^	0.136^a^	0.157^b^	58.0^ef^	89.6^bc^
	Salinity	3.37^bcd^	4.37^ab^	2.04^f^	1.56^bcd^	0.120^a–d^	0.116^cd^	0.100^fgh^	0.154^bc^	50.0^gh^	69.3^gh^
	Alkalinity	3.80^bc^	4.30^ab^	2.77^a–e^	1.30^de^	0.110^cde^	0.106^d^	0.104^efg^	0.122^fgh^	46.6^h^	71.3^fg^
Ambient light	Control	3.09^cde^	3.43^cde^	2.54^b–f^	1.76^abc^	0.136^a^	0.106^d^	0.120^b^	0.126^efg^	51.6^fgh^	66.3^gh^
	Salinity	5.52^a^	4.81^a^	2.24^ef^	1.63^a–d^	0.110^cde^	0.100^de^	0.092^h^	0.076^i^	34.3^i^	63.0^hi^
	Alkalinity	3.99^b^	4.77^a^	2.51^b–f^	1.56^bcd^	0.103^de^	0.076^e^	0.105^def^	0.111^h^	30.6^i^	59.3^i^
Significance	Light (L)	***	*	ns	**	ns	***	***	***	***	***
	Stress (S)	***	***	ns	**	***	***	***	***	***	***
	L × S	**	ns	*	ns	ns	*	***	***	**	***

Values are means ± SE of three replicates. Bars with different letters show significant differences at P ≤ 0.05 (Duncan).

Significance according to ANOVA, ns, *, **, ***, no significant and significant P ≤ 0.05, 0.01, 0.001, respectively.

Control (no stress), salinity (80 mM NaCl) and alkalinity (40 mM NaHCO_3_).

Photon flux density (PPFD) 200 ± 10 mmol_m^–2^ s^–1^.

SAS software version 9.4 was used for data analysis.

### Correlation analysis

**Figure 4:**
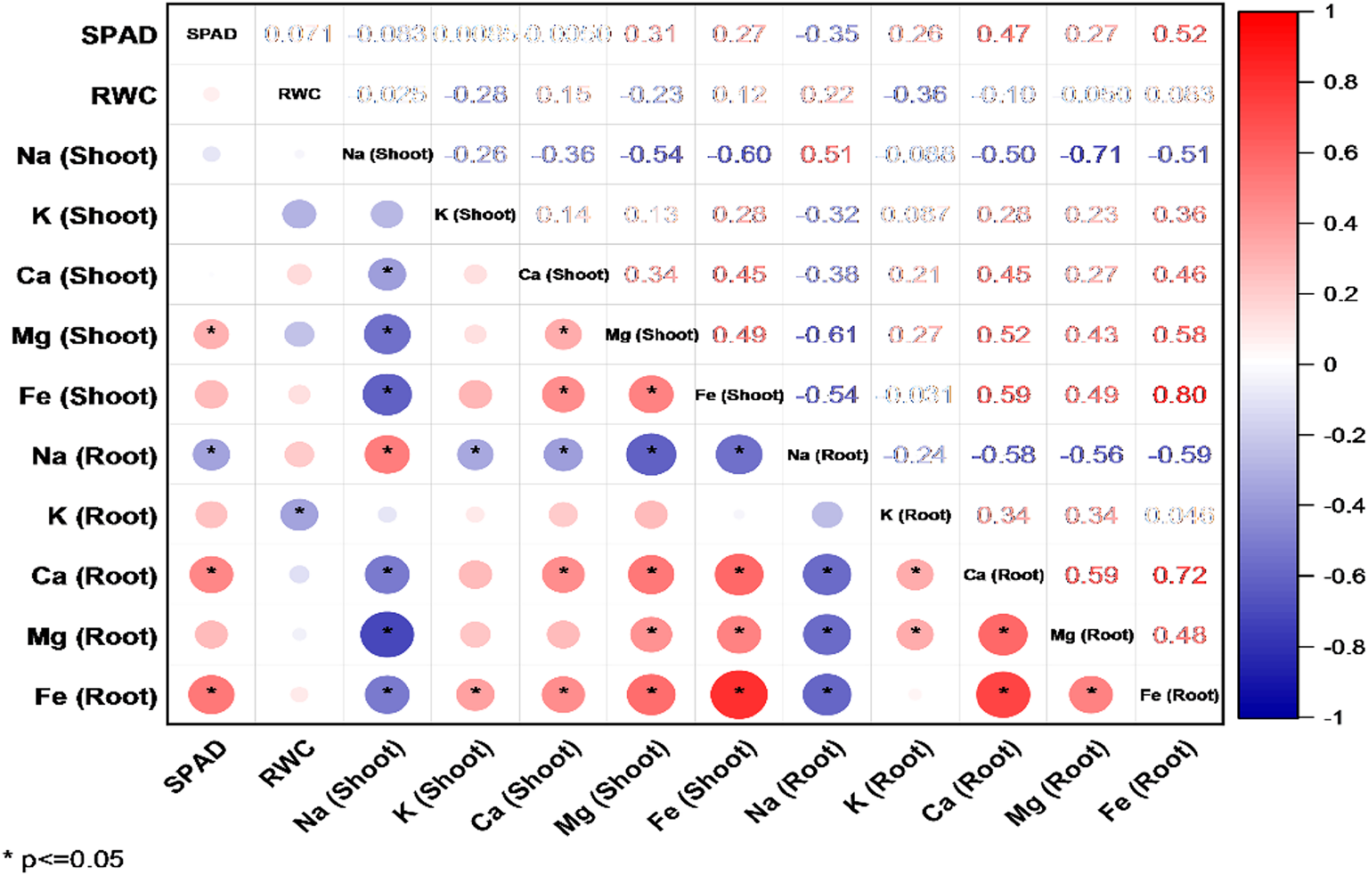
Correlation plot between SPAD, RWC, and shoot and root elements. The size and color intensity of circles are proportional to Pearson’s correlation coefficient at P < 0.01. Red circles indicate positive correlations, while blue are negative correlations. In the correlogram scale from − 1 to + 1, Pearson’s correlation coefficient for variables is on the vertical and horizontal axis. *Indicates values that are statistically different at P < 0.05. A correlation plot was drawn with Origin Pro software version 2021.

The correlation plot (Fig. 4) shows the correlations between SPAD, RWC, and the shoot and root elements. The results show that the SPAD index was not significantly correlated with RWC, leaf Na, K, and Ca. The SPAD index had a positive correlation with leaf Mg and Fe and root K, Ca, Mg, and Fe. The SPAD index had a negative correlation with root Na. The RWC index was negatively correlated with the shoot and root K and was unrelated to other variables.

### Principal component analysis

**Figure 5:**
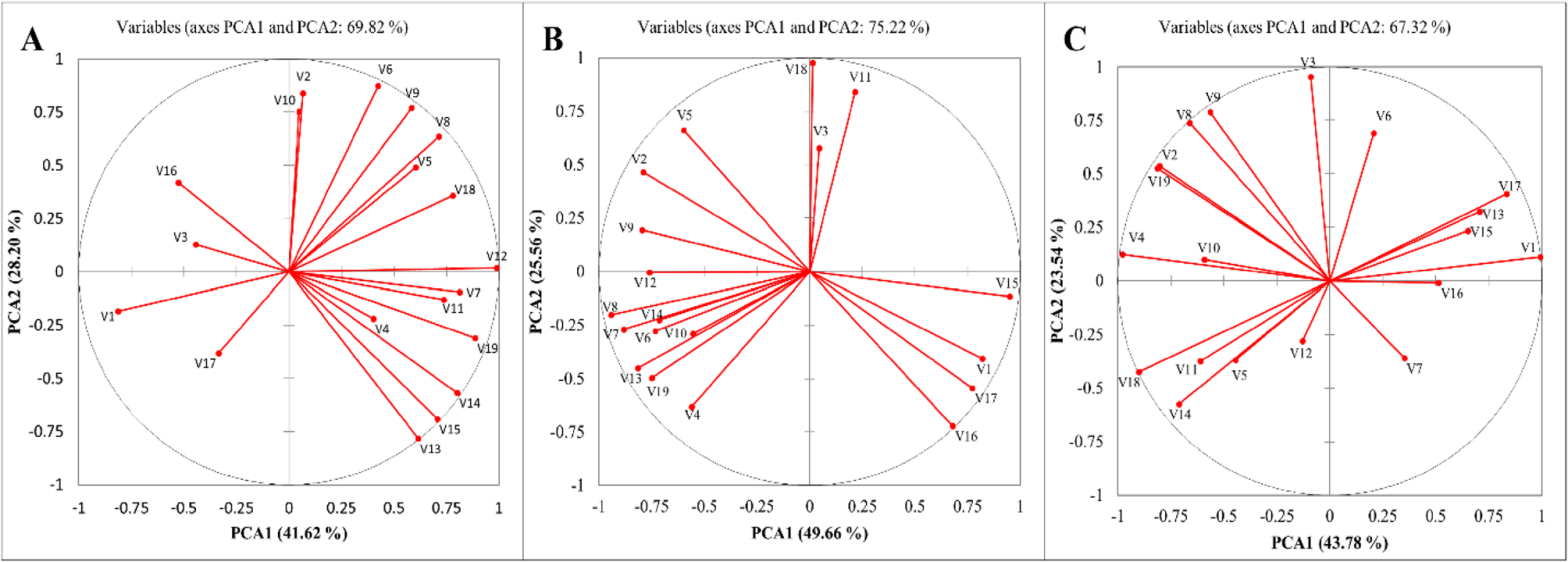
Vector graphs show the relative “contribution” of each input variable to the formation of the principal components. Principle component biplot based on variations of five levels of light spectra under (**A**) control (non-stress); (**B**) salinity stress; (**C**) alkalinity stress. Principal component analysis and biplots were performed using XLSTAT software version 2015. V1: Na leaves; V2: Fe leaves: V3: K leaves; V4: Ca leaves; V5: Mg leaves; V6: Inflorescence; V7: Early yield; V8: Fruit number; V9: Soluble solids; V10: a; V11: Hue; V12: Leaf dry weight; V13: Root dry weight; V14: Green leaves; V15: Chlorotic leaves; V16: Tip burned leaves; V17: Dry leaves; V18: SPAD; V19: RWC.

Vector plots (Fig. 5) show the relative participation of each variable in the formation of the principal components (PCA 1 and PCA 2). Vector size indicates the effect of each variable, and the direction of the vector depends on the values of PCA 1 and PCA 2. These parameters had different sensitivities to stress and contributed differently to the formation of the principal components. First, the data were standardized with a mean of zero and a unit variance. PCA was performed to summarize the change in 19 parameters in five optical spectrum treatments and three stress levels separately. In the control treatment, PCA explained 69.82% of the total changes in the five light spectrum treatments (Fig. 5A). This value was 75.22% and 67.32% for the salinity and alkalinity treatments, respectively (Fig. 5B,C). Regardless of the direction of the effect, parameters V2 (Fe leaves) and v10 (a) had the lowest contribution to the first principal component of the changes caused by the five light spectrum treatments in the control treatment. These parameters were V3 (P-leaves), V11 (hue), and V18 (SPAD) in the salt stress treatment and V3 (P-leaves), V6 (inflorescence), and V12 (dry leaf weight) in the alkali stress treatment. Of these parameters, V12 (dry leaf weight) in the control treatment and V15 (chlorotic leaves) in the salt stress treatment, and V1 (Na leaves) in the alkalinity stress treatment had the largest contribution to the first principal component.

## Discussion

The first response of the plant to salinity stress is leaf area limitation and low growth^32^. The decrease in strawberry yield under salinity stress would be due to a reduction in the number and weight of fruits. In addition, salt stress leads to leaf chlorosis, a decrease in leaf area, and reduced photosynthesis due to chlorophyll degradation^33^. Figure 5 shows that chlorotic leaves had the highest proportion of variables under salt stress. The correlation of the traits showed that salt and alkalinity stress decreased the dry weight of roots and leaves by increasing the number of chlorotic and tip burned leaves. It has been shown that under the influence of salinity and alkalinity, there is a decrease in the relative amount of leaf water^30,34^. The accumulation of salt in the root zone prevents roots from absorbing water by reducing the osmotic potential, thus decreasing the water content of the leaf^35^. Under salt and alkalinity stress, plants accumulate inorganic ions in the vacuole to lower the water potential of the cell. Under alkaline stress, there is a decrease in water content due to the deleterious effect of high pH on plant roots and water uptake or solute accumulation^36^. It has been reported that soluble solids content is significantly reduced in strawberry cultivars with salinity and alkalinity^30,33^. Yang et al.^37^ suggested that the decrease in soluble sugar content under alkaline stress was due to high pH and abnormal metabolism due to intercellular ion imbalance and damage to root function caused by alkaline stress.

Plants can receive and sense changes in light quality through light receptors and regulate their growth and development through signaling pathways. It is well established that the morphological, physiological and nutritional quality of plants is affected by the quality and intensity of light^38^. Chloroplasts absorb and use mainly blue and red light for photosynthesis^39^. Our study investigated the effect of some light spectra as supplementary light on vegetative and reproductive process of strawberry cv. Camarosa. Some light spectra have been shown to help plants become more resistant to biological and abiotic stresses^40^. Our results showed that blue and red light, especially blue light, had a stronger effect on vegetative traits and could reduce stress more than other light spectra. Blue light is essential for chlorophyll biosynthesis. In addition, several studies have shown that the combination of blue and red spectrum plays an important role in leaf area and plant biomass^14,41^. It is found that light quality can affect fruit size^42^. In other studies, a combination of blue and red light was used, which significantly increased fruit production^43^. Wang et al.^44^ reported that shoot dry weight increased under blue/red light. This increase was attributed to the effect of blue/red light on leaf number and leaf area. Our results showed that under stress conditions, blue/red light caused a significant increase in leaf area compared to other light spectra. Experiments show that blue and red LED light increases vegetative and reproductive traits of plants under greenhouse conditions^45^. Previous studies reported greater leaf area of lettuce under red and blue/red light^14^. Our results also showed that blue/red light under salt stress and blue light under alkaline stress caused the highest increase in leaf area. These results can show the importance of using these light spectra on plant growth under stress conditions.

Under stress conditions, plants grown under blue light had the highest SPAD index. In our other experimental results, it was shown that under stress conditions, the spectra of blue and red light and their combination affected the gas exchange parameters of plants. These spectra increased the CO_2_ uptake rate (A) and water use efficiency (WUEi)^46^. It can be concluded that blue and red light improves the resilience of plants to stress conditions by affecting the photosynthetic efficiency of the plant. Blue light has been shown to stimulate pigment biosynthesis^47^. Previous studies have shown that a lack of blue light inhibits chlorophyll biosynthesis in pink hybrids^48^ and cucumber seedlings^19^. Under alkaline stress conditions, blue/red light increased RWC by increasing potassium uptake, which may be due to the role of K in stomatal regulation and osmosis in the plant^49^.

One of the most important criteria for evaluating strawberry fruit quality is the fruit color index^50^. Anthocyanins belong to phenolic compounds and valuable antioxidant compounds that increase the tolerance of plants to salt stress^51^. It has also been reported that large amounts of anthocyanins accumulate in response to the red light spectrum^52^. According to our results, red light could increase a index under alkaline stress conditions, which was not significantly different from salt stress. The results showed that blue light had a significant effect on croma and hue index. Blue light is one of the effective factors to increase anthocyanin biosynthesis in fruits^53^. The synthesis of anthocyanins under blue added light in strawberry fruits during ripening has been reported^54^.

Bicarbonate ions interfere with the uptake and transport of essential plant nutrients^55^. In this study, the elements Fe, Ca, K, and Mg was reduced under alkaline stress. As Fig. 5 shows, sodium ions had the greatest effect under alkaline stress conditions. Na ion is the major toxic ion in saline soils. Low Na and high K levels in the cytoplasm are essential for maintaining the activity of some enzymes^56^. Plants subjected to salinity and alkalinity stress inevitably accumulate large amounts of sodium. Increasing sodium prevents the plant from absorbing potassium and decreasing the potassium content in the plant^57^. One of the most important roles of K is water balance and solute transport in the woody vessels of plants^55^. It has been shown that alkaline stress can severely affect K uptake by rice roots^49^. Potassium plays an important role in solute transport in the xylem and water balance of plants^58^. Plants exposed to salinity and alkaline stress take up large amounts of sodium, which prevents the uptake of K and reduces the K content in the plant^57^. Our results showed that blue/red light under stress increased potassium content compared to the control. Sun et al.^58^ reported that plant uptake and accumulation of K under salt stress increased plant resistance. The transport of K, Ca, and Mg is disrupted by Na under salinity conditions and may interfere with plant metabolism and reduce plant growth^59^.

## Conclusions

Analysis of the morphological, physiological, and elemental characteristics of strawberry plants shows that plants adopt different strategies against abiotic stress depending on light quality. The results showed that blue and red light spectra affect the absorption of elements and photosynthetic apparatus of plants. Therefore, they improve vegetative and reproductive growth and increase plant resistance to stress. Although white/yellow light improves vegetative traits of strawberry plants under non-stress conditions, plants exposed to additional blue, red, and especially the combination of blue and red light tolerate stress conditions better under stress conditions. Light spectra affect the resistance of plants to stress conditions by affecting the absorption of elements and the photosynthetic apparatus performance. Understanding the effects of these spectra under different growing conditions provides a basis for manipulating light spectra to improve plant resistance to stress conditions. LED technology may be promising for greenhouse plants, but more studies need to be conducted on the effects of LEDs on different plants and under other conditions. Incidence spectrum and photon flux density are the two main factors in determining the proper light of a plant that control plant growth in response to light conditions. It can be concluded that light quality can affect many aspects of strawberry plant morphology and physiology. Therefore, the optimal growth of a species or cultivar requires a certain amount and quality of light.

## Data Availability

The data presented in this study are available on request from the corresponding authors. The data are not public.
